# Artemisinin Attenuates Lipopolysaccharide-Stimulated Proinflammatory Responses by Inhibiting NF-κB Pathway in Microglia Cells

**DOI:** 10.1371/journal.pone.0035125

**Published:** 2012-04-13

**Authors:** Cansheng Zhu, Zhaojun Xiong, Xiaohong Chen, Fuhua Peng, Xueqiang Hu, Yanming Chen, Qing Wang

**Affiliations:** 1 Department of Neurology, The Third Affiliated Hospital, Sun Yat-Sen University, Guangzhou, China; 2 Department of Cardiovascular Diseases, The Third Affiliated Hospital, Sun Yat-Sen University, Guangzhou, China; 3 Department of Metabolism and Endocrinology, The Third Affiliated Hospital, Sun Yat-Sen University, Guangzhou, China; Virginia Commonwealth University, United States of America

## Abstract

Microglial activation plays an important role in neuroinflammation, which contributes to neuronal damage, and inhibition of microglial activation may have therapeutic benefits that could alleviate the progression of neurodegeneration. Recent studies have indicated that the antimalarial agent artemisinin has the ability to inhibit NF-κB activation. In this study, the inhibitory effects of artemisinin on the production of proinflammatory mediators were investigated in lipopolysaccharide (LPS)-stimulated primary microglia. Our results show that artemisinin significantly inhibited LPS-induced production of tumor necrosis factor-alpha (TNF-α), interleukin-6 (IL-6), monocyte chemotactic protein-1 (MCP-1) and nitric oxide (NO). Artemisinin significantly decreased both the mRNA and the protein levels of these pro-inflammatory cytokines and inducible nitric oxide synthase (iNOS) and increased the protein levels of IκB-α, which forms a cytoplasmic inactive complex with the p65-p50 heterodimeric complex. Artemisinin treatment significantly inhibited basal and LPS-induced migration of BV-2 microglia. Electrophoretic mobility shift assays revealed increased NF-κB binding activity in LPS-stimulated primary microglia, and this increase could be prevented by artemisinin. The inhibitory effects of artemisinin on LPS-stimulated microglia were blocked after IκB-α was silenced with IκB-α siRNA. Our results suggest that artemisinin is able to inhibit neuroinflammation by interfering with NF-κB signaling. The data provide direct evidence of the potential application of artemisinin for the treatment of neuroinflammatory diseases.

## Introduction

Microglia, which are the resident macrophages of the central nervous system (CNS), are recognized as the primary component of the brain immune system [1]. They are activated during neuropathological conditions to restore CNS homeostasis [2]. Once activated, microglia undergo morphological changes, proliferate and upregulate surface molecules [3]. Activated microglia can promote neuronal injury through the release of proinflammatory and cytotoxic factors, including tumor necrosis factor (TNF)-α, interleukin (IL)-1β, IL-6, NO and reactive oxygen species (ROS) [4]. Studies have demonstrated that the inhibition of pro-inflammatory mediators in microglia can attenuate the severity of Alzheimer's disease (AD), Parkinson's disease (PD), trauma, multiple sclerosis (MS) and cerebral ischemia [5]–[9]. Thus, anti-inflammatory treatment via inhibition of microglial activation is regarded as a promising strategy for the prevention of neurodegenerative diseases [10].

Artemisinin (qinghaosu) is the active component of *Artemisia annua* L. and is approved worldwide for the treatment and prevention of malaria [11]. In addition to its antimalarial properties, artemisinin and its derivatives have been demonstrated to affect other cellular biochemical processes [12], [13], such as proliferation, angiogenesis, apoptosis and oxidative stress. Recently, artemisinin has been shown to exert an inhibitory effect on inducible nitric oxide synthase (iNOS) synthesis and NF-κB activation in human astrocytoma T67 cells [14]. A derivative of artemisinin, SM933, has been found to inhibit the activity of NF-κB by preventing its degradation via upregulation of its inhibitory protein kappa B alpha (IκB-α) in MOG-reactive splenocytes [15]. Taken together, these studies support the conclusion that artemisinin may play a role in immune regulation and act to reduce inflammation. The anti-inflammatory effects of artemisinin on microglial activation, however, are unknown. In the present study, we investigated the effects of artemisinin on lipopolysaccharide (LPS)-stimulated pro-inflammatory responses in microglia and the signaling mechanism by which artemisinin modulates the pro-inflammatory response.

## Results

### Artemisinin is not toxic to primary rat microglia in the culture conditions used

**Figure 1:**
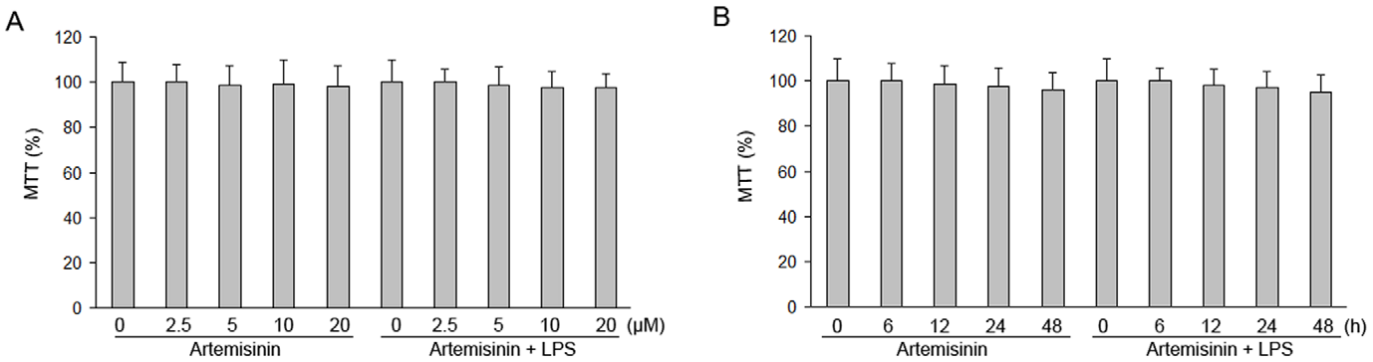
Effect of artemisinin and LPS on primary rat microglia viability. Viability in artemisinin-treated cells was evaluated using the MTT assay. Cells were incubated with artemisinin (2.5 to 20 µM) and LPS (1 µg/ml) for 1 h and with 10 µM artemisinin and 1 µg/ml LPS for 6 to 48 h. The results are displayed as the percentages of the control samples. The data are presented as the means ± S.E.M. (n = 5) for three independent experiments.

The cytotoxic effects of artemisinin were evaluated with the MTT assay by measuring the viability of primary rat microglia that were incubated with artemisinin (2.5, 5, 10, or 20 µM) for 1 h in the presence or absence of LPS (1 µg/ml). We also examined the viability of primary rat microglia treated with 10 µM artemisinin for 6, 12, or 48 h in the presence or absence of LPS (1 µg/ml). Interestingly, no significant differences in cell viability were found between normal primary rat microglia and microglia treated with 10 µM artemisinin for 48 h (Fig. 1), which indicates that the inhibitory effect that we observed was not due to cytotoxicity.

### Artemisinin inhibits NO and iNOS production in LPS-stimulated primary rat microglia

**Figure 2:**
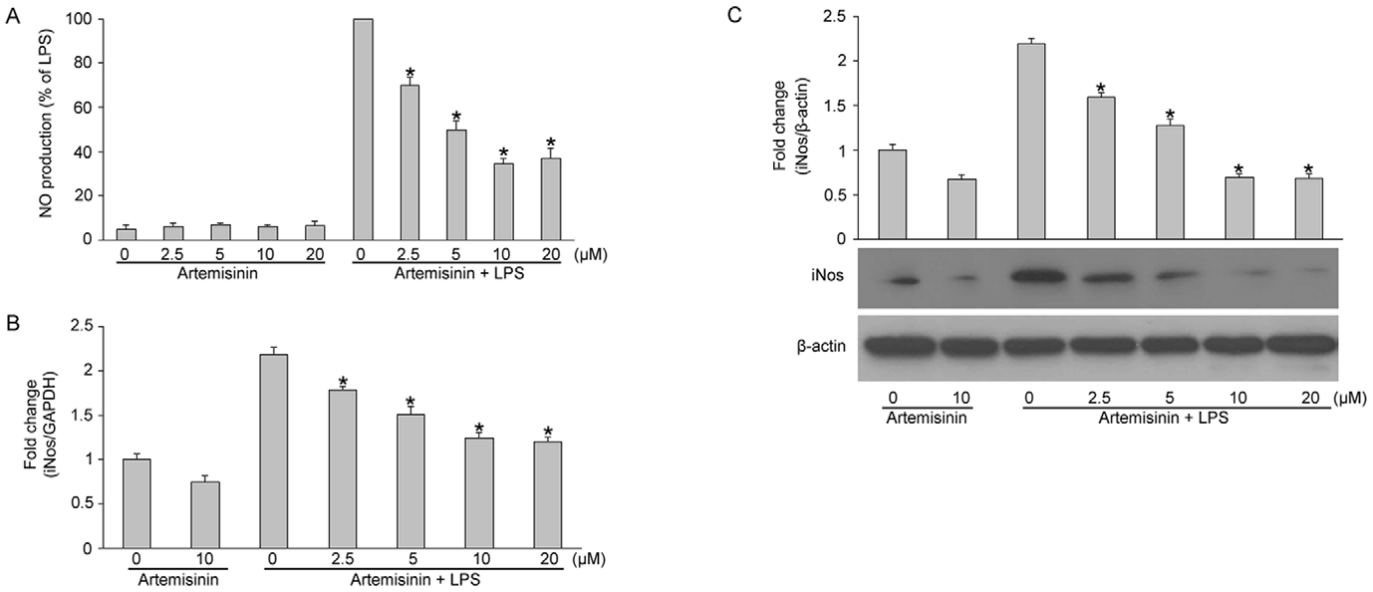
Artemisinin inhibition of LPS-induced nitrite production in primary rat microglia. Cells were pretreated with the indicated concentrations of artemisinin for 1 h before incubating with LPS (1 µg/ml) for 3 h. The culture medium was collected to assay the nitrite levels (A). The cells were lysed, and the lysates were prepared for the detection of mRNA levels (B) and western blot analysis with an anti-iNOS antibody (C). The data are presented as the means ± S.E.M. (n = 5) for three independent experiments. *P<0.05 vs. LPS alone.

To investigate the effects of artemisinin on NO production in LPS-stimulated primary rat microglia, cells were treated with LPS alone or with various concentrations of artemisinin for 24 h. As shown in Fig. 2A, LPS alone markedly induced NO production compared with the control group (P<0.01); however, artemisinin significantly reduced LPS-induced NO production in a dose-dependent manner. Pretreatment of microglia with 2.5, 5, 10, or 20 µM artemisinin for 1 h prior to LPS stimulation decreased NO production to 70.0±3.6%, 49.7±4.0%, 34.7±2.5%, and 37.0±4.6% (P<0.05 vs. the LPS group), respectively, and the maximal inhibitory efficacy occurred at 10 µM (P>0.05 vs. the 20 µM group). The effect of artemisinin on iNOS mRNA expression was measured using RT-PCR analysis. Although the iNOS mRNA was barely detected in unstimulated primary rat microglia, it was expressed at high levels following stimulation with 1 µg/mL LPS for 3 h. Pretreatment with artemisinin inhibited this LPS-stimulated iNOS mRNA production in a dose-dependent manner (Fig. 2B). The protein levels of iNOS that were detected by western blotting were also repressed by artemisinin (Fig. 2C). These results show that artemisinin inhibited NO production through the downregulation of iNOS mRNA and protein expression in LPS-stimulated primary microglia.

### Artemisinin inhibits the migration of microglia

**Figure 3:**
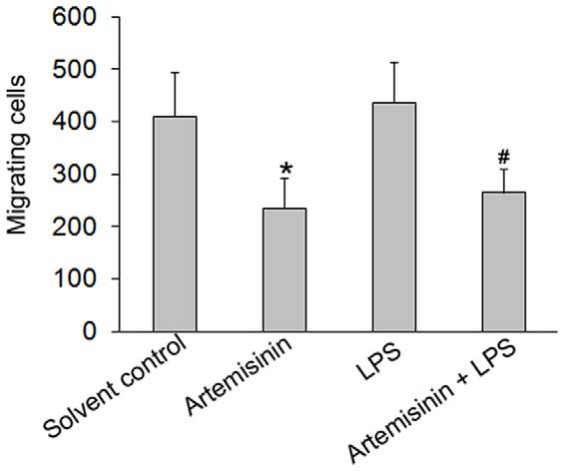
Artemisinin inhibition of microglial motility. Transwell chamber migration of BV-2 microglial cells treated with solvent control, 10 µM artemisinin, 100 ng/ml LPS, or 10 µM artemisinin + 100 ng/ml LPS for 24 hours. The absolute number of migrating cells was counted in the lower chamber and mean values ± SEM are displayed. * P≤0.05 for artemisinin vs. control, and # P≤0.05 for artemisinin + LPS vs. LPS, Student's t test.

In order to measure microglial cell motility and to study the effects of artemisinin, we performed transwell migration assays. BV-2 cells (a mouse microglial cell line) were added to the upper well, and LPS, artemisinin, LPS + artemisinin, or DMSO as solvent control were added to the lower chamber medium. The migratory capacity of BV-2 cells was not changed by the activation agent LPS alone (Fig. 3). In both, the resting and the activated microglial phenotype, artemisinin caused a significant attenuation of microglial migration (Fig. 3). These results indicate that artemisinin has an inhibitory effect on microglial motility.

### Artemisinin inhibits the expression of cytokines in LPS-stimulated primary rat microglia

**Figure 4:**
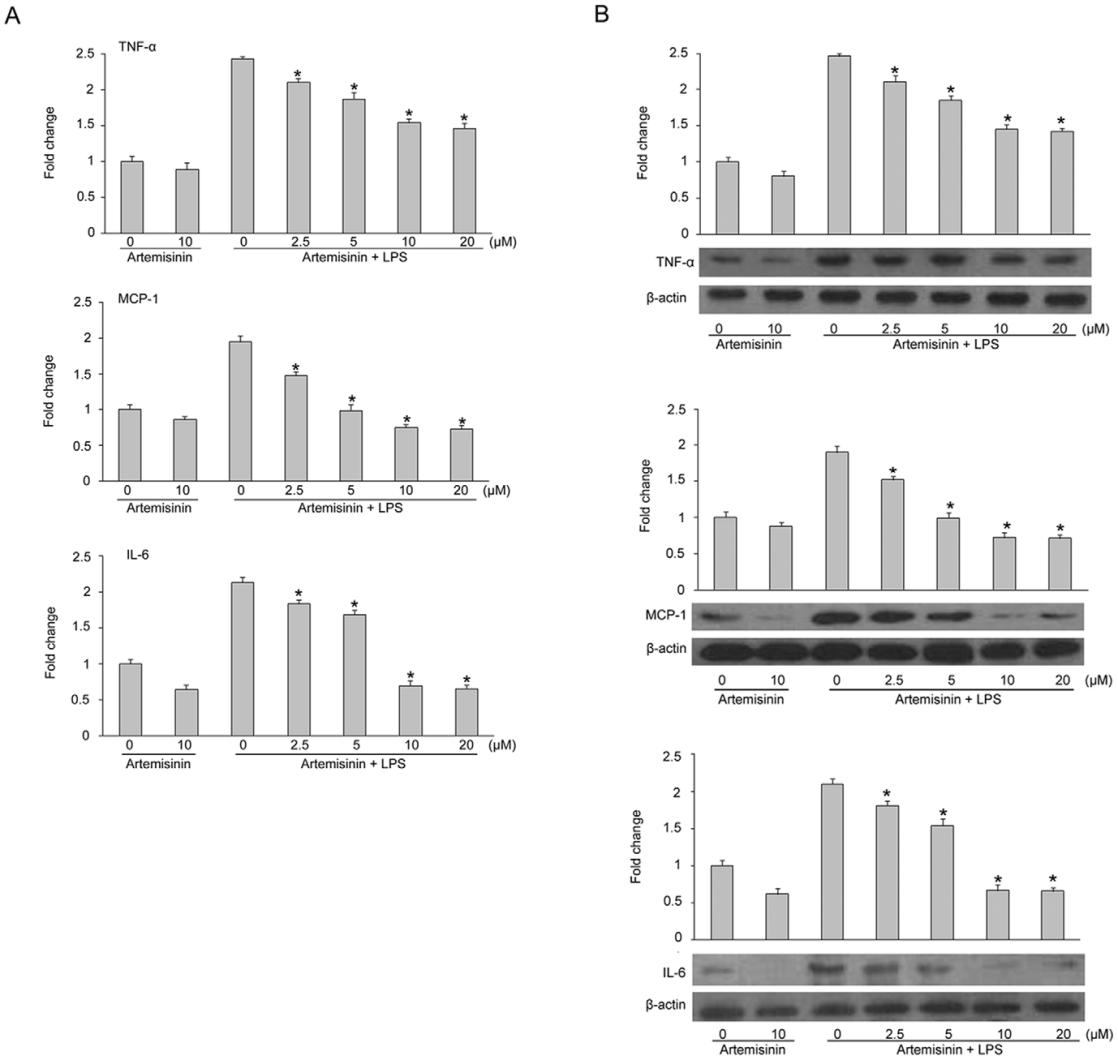
The effects of artemisinin on LPS-induced TNF-α, MCP-1 and IL-6 expression in primary rat microglia. Cells were treated with the indicated doses of artemisinin for 1 h before LPS treatment (1 µg/ml). After incubation for 3 h, the mRNA levels of TNF-α, MCP-1 and IL-6 were determined by RT-PCR (A) and the protein levels were analyzed by western blot (B). The data are presented as the means ± S.E.M. (n = 5) for three independent experiments. *P<0.05 vs. LPS alone.

To elucidate the potential effects of artemisinin on pro-inflammatory cytokine production (e.g., TNF-α, MCP-1 and IL-6), primary rat microglia were incubated with artemisinin (2.5, 5.0, 10, and 20.0 µM) in the presence or absence of LPS (1 µg/ml). RT-PCR analysis was used to examine the cytokine mRNA expression levels, and the expression levels of TNF-α, MCP-1 and IL-6 were significantly up-regulated after treatment with LPS (1 µg/ml). Interestingly, the LPS-stimulated mRNA levels of the pro-inflammatory cytokines TNF-α, MCP-1 and IL-6 were reduced by artemisinin (Fig. 4A). Moreover, the protein levels of TNF-α, MCP-1 and IL-6 in the LPS-stimulated primary rat microglia were significantly reduced dose responsively by artemisinin treatment (Fig. 4B). These suggested that artemisinin negatively regulates the expression of TNF-α, MCP-1 and IL-6 in LPS-stimulated primary microglia.

### Artemisinin inhibits LPS-induced NF-kB activation in primary microglia

**Figure 5:**
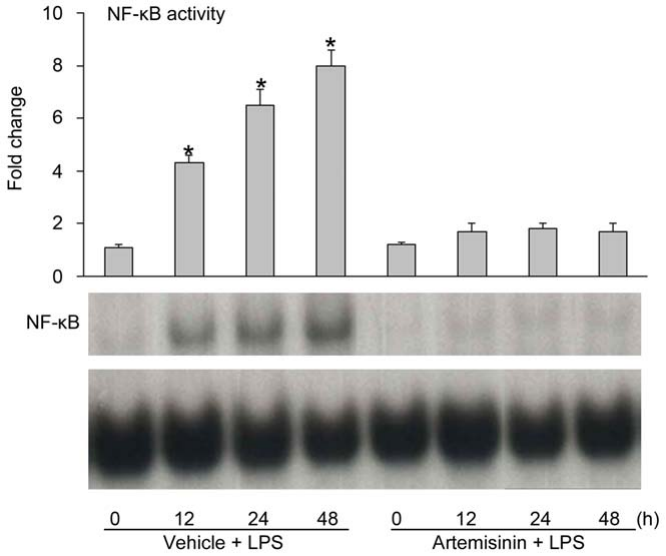
Artemisinin inhibits LPS-induced NF-kB activation in primary microglia. The DNA binding activity of NF-κB was evaluated by EMSA in cells pretreated with 10 µM artemisinin for 12, 24, or 48 h prior to a 3 h incubation with LPS (1 µg/ml). The data represent the means ± S.E.M of three independent experiments. * P<0.05 vs. the artemisinin group.

Previous studies have shown that LPS increases NF-κB subunit activation (via phosphorylation, ubiquitination, degradation and translocation of p65 and IκB-α) and regulates the expression of iNOS and pro-inflammatory cytokines [16]–[18]. Activation of NF-κB has been reported to be inhibited by artemisinin [14], [19]. We performed EMSA (electrophoretic mobility shift assay) studies to investigate whether artemisinin inhibited NF-κB activation in LPS-stimulated primary microglia. Interestingly, artemisinin abolished the increased DNA binding activity of NF-κB that was observed in primary microglia stimulated with LPS (Fig. 5). These data demonstrate that artemisinin inhibits NF-κB activation in LPS-stimulated primary microglia and that this mechanism may contribute to the anti-inflammatory effect of artemisinin.

### Artemisinin treatment increases the IκB-α level in primary microglia stimulated with LPS

**Figure 6:**
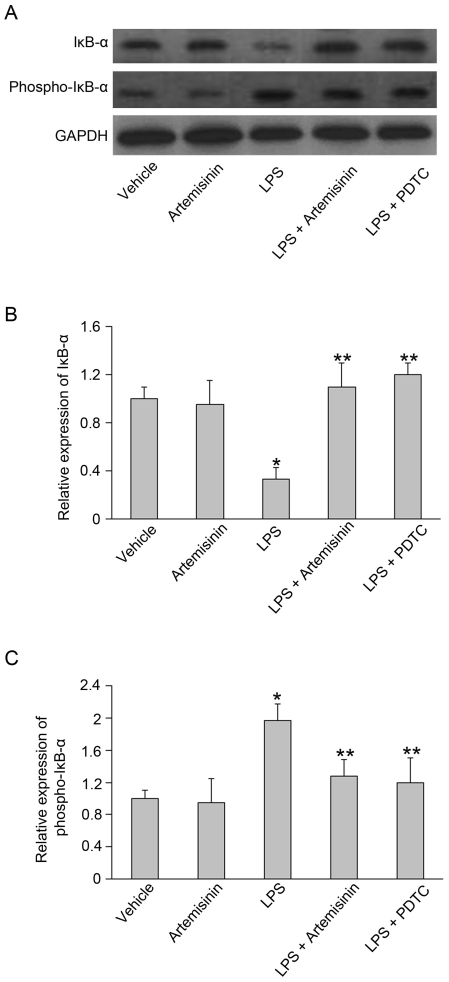
The effects of artemisinin on LPS-induced degradation and phosphorylation of IκB-α. (A) After treatment with artemisinin (10 µM) or PDTC (a specific inhibitor of NF-κB, 300 µM) for 24 h, primary rat microglia were stimulated with LPS (1 µg/ml) for 30 min. The LPS-induced degradation and phosphorylation of IκB-α were analyzed by western blot analysis. (B and C) Data are presented as the means ± S.E.M. from five separate experiments. *P<0.05 vs. untreated control and **P<0.05 vs. treatment with LPS alone.

Inhibition of NF-κB signaling may occur through a variety of mechanisms, one of which may involve the enhanced expression of IκB-α, which forms an inactive cytoplasmic complex with the p65-p50 heterodimeric complex. We determined the protein levels of IκB-α by western blot analysis using specific anti-IκB-α and anti-phosphorylated IκB-α antibodies. Interestingly, the addition of artemisinin to primary microglia exposed to LPS resulted in an increase in the level of IκB-α. Because activation of NF-κB requires IκB-α degradation, we assessed whether artemisinin affected the protein level of phosphorylated IκB-α. Our data show that the protein level of IκB-α was reduced (Fig. 6B and C), but this reduction was not observed when cells were co-incubated with artemisinin. In addition, we compared artemisinin to PDTC, which is a specific inhibitor of NF-κB, to investigate whether artemisinin was inhibiting NF-κB activity. Figure 6 shows arteminisin (10 µM) alone had no effect on the IκB-α degradation and phosphorylation; however, it prevented LPS-induced degradation and phosphorylation of IκB-α. Artemisinin inhibited NF-κB activity to a similar degree as PDTC (300 µM) (Fig. 6A–C). These results indicate that artemisinin inhibited LPS-induced NF-κB activation by preventing IκB-α degradation and phosphorylation in primary microglia.

### The anti-inflammatory effects of artemisinin in LPS-stimulated primary microglia were blocked by siRNA

**Figure 7:**
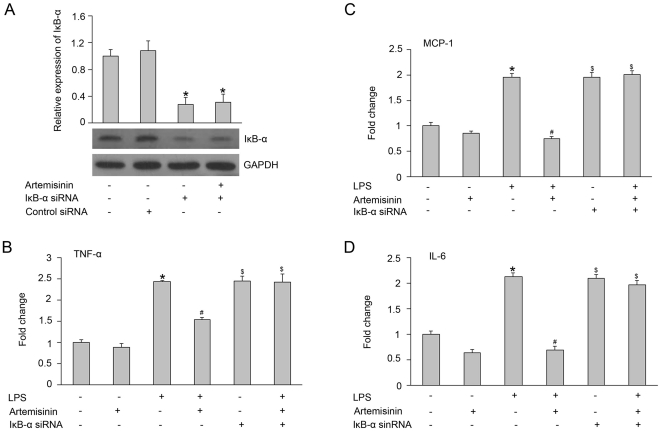
LPS-induced TNF-α, MCP-1 and IL-6 mRNA expression in primary rat microglia after treatment with IκB-α siRNA. IκB-α protein expression in primary rat microglia transfected with IκB-α siRNA for 48 h (A). The effects of artemisinin on LPS-induced TNF-α (B), MCP-1 (C) and IL-6 (D) mRNA expression in cultured cells after incubation with IκB-α siRNA for 48 h. The data are presented as the means±S.E.M. from five separate experiments. *P<0.05 vs. control, ^#^P<0.05 vs. treatment with LPS alone, and ^$^P<0.05 vs. treatment with LPS and artemisinin.

To determine whether the anti-inflammatory effects of artemisinin are due to the inhibition of NF-κB activation by the enhanced expression of IκB-α, we also examined the changes in cytokine expression levels in LPS-stimulated primary rat microglia after IκB-α siRNA transfection. We observed that silencing IκB-α with siRNA increased the TNF-α, MCP-1 and IL-6 mRNA expression levels in LPS-stimulated primary microglia. Interestingly, artemisinin inhibited the LPS-induced increase in TNF-α, MCP-1 and IL-6 mRNA expression, but this effect was significantly reduced after IκB-α siRNA transfection (Fig. 7A–D).

## Discussion

To the best of our knowledge, the present study was the first to demonstrate that artemisinin inhibits the inflammatory activation of microglia. Artemisinin significantly reduced inflammatory mediators, such as TNF-α, MCP-1, IL-6 and NO production, in LPS-activated primary microglia *in vitro*, and the RT-PCR analysis showed that artemisinin markedly suppressed TNF-α, MCP-1, IL-6 and iNOS gene expression. The anti-inflammatory properties of artemisinin are mediated by the interruption of NF-κB signaling pathways in microglia. Artemisinin and its derivatives are commonly used in the treatment of patients with severe and chloroquine-resistant malaria [20]. To the best of our knowledge, however, there is no evidence regarding the effects of artemisinin on the inflammatory activation of microglia. Our study found that treatment with 20 µM artemisinin for 48 h did not affect cell viability, which indicates that the primary effects of artemisinin actions are anti-inflammatory rather than cytotoxic.

Microglial cells are brain macrophages that serve specific functions in the defense of the CNS against microorganisms, the removal of tissue debris in neurodegenerative diseases or during normal development, and in autoimmune inflammatory disorders of the brain [3]. Microglia activation plays a crucial role in the initiation and progression of brain inflammation, which is thought to aggravate pathological conditions in the CNS by releasing various proinflammatory cytokines and free radicals [21]–[23]. These factors are believed to contribute to microglia-mediated neurodegeneration [22], [24], [25]. Several immune stimulants, such as IFN-γ, LPS and TNF-α, are known to activate microglial cells [26]–[28]. LPS, which is a natural Toll-like receptor 4 (TLR4) ligand, has been the most extensively utilized microglial activator for the induction of an inflammatory response. Thus, the present study used LPS as an experimental model to activate microglia.

Activated microglia produce a wide range of proinflammatory mediators, including TNF-α, IL-6, IL-1β, ROS, and NO. NF-κB is an essential and ubiquitous transcription factor for the expression of many inflammation-related genes, including TNF-α, IL-6, IL-1β and iNOS. LPS has also been reported to activate NF-κB in microglia [29], [30], and NF-κB activation and IκB-α degradation are known to be involved in LPS/IFNγ-induced iNOS expression [16]. Recent studies have indicated that artemisinin and its derivatives may exert anti-inflammatory and immunoregulatory effects by inhibiting NF-κB activation [19]. In human rheumatoid arthritis fibroblast-like synoviocytes, artesunate, an artemisinin derivative, has the ability to inhibit TNF-α- induced production of proinflammatory cytokines via inhibition of NF-κB and PI3 kinase/Akt signal pathway [19]. Both in THP-1 and in RAW 264.7 macrophages, artemisinin and its derivatives inhibited pro-inflammatory cytokine production by NF-κB pathway [31], [32]. Due to its anti-inflammatory properties, the role of artemisinin in LPS-activated microglia was investigated in this study. Our results showed that LPS mediated an intense proinflammatory response in primary rat microglia, which was consistent with numerous reports. The production of NO free radicals and pro-inflammatory cytokines, including TNF-α, IL-6, and MCP-1, were increased in the LPS-stimulated microglia after 24 h. Interestingly, concentrations of artemisinin ranging from 1 to 20 µM significantly suppressed microglial activation and the pro-inflammatory response induced by LPS in a concentration-dependent manner without causing cytotoxicity. Artemisinin inhibited the activity of NF-κB and its capacity to bind DNA in primary microglia subjected to LPS. NF-κB is a critical signal transcription factor for regulating immune and inflammatory responses. Importantly, the activity of NF-κB is regulated by its subcellular localization, and NF-κB is retained in the cytosol when bound to inhibitors of κB (i.e., IκB). In resting cells, NF-κB is sequestered in the cytoplasm by the IκB family, including IκB-α and IκB-β. Activation of the IκB proteins, which can be induced by a variety of stimuli, such as pro-inflammatory cytokines, results in the phosphorylation of the IκB proteins by a complex of IκB kinases (IKK). After phosphorylation, IκB proteins are ubiquitinated and rapidly degraded by the proteasome, which allows NF-κB to be released from IκB and translocate to the nucleus where it can initiate transcription by binding to numerous specific gene promoter elements [33], [34]. In the present study, we demonstrated that artemisinin increased the expression of IκB-α. The effect of artemisinin on activated microglia was blocked by IκB-α siRNA. Therefore, increased expression of IκB-α likely resulted in persistent binding to NF-κB, which blocked NF-κB translocation to the nucleus and prevented its activity.

In conclusion, the present study demonstrated that artemisinin inhibited LPS-induced production of inflammatory mediators, such as TNF-α, IL-6, MCP-1, and NO, in primary microglia. The anti-inflammatory properties of artemisinin are mediated by its effects on the NF-κB signaling pathway in LPS-stimulated microglia. The data from the present study suggest that artemisinin may be developed as a therapeutic agent for the treatment of neuroinflammatory diseases that are characterized by excessive microglial activation.

## Materials and Methods

### Chemicals and Reagents

Griess reagent, LPS, N,N-dimethyl sulfoxide (DMSO) and pyrrolidinedithiocarbamate (PDTC) were purchased from Sigma-Aldrich (St. Louis, MO, USA). Dulbecco's modified Eagle's medium (DMEM/F12), fetal bovine serum (FBS), antibiotics, trypsin-EDTA, phosphate-buffered saline (PBS) and other products for cell culture were purchased from Invitrogen (Carlsbad, CA, USA). Antibodies against IκB-α and phosphorylated-IκB-α (Ser32) were purchased from Santa Cruz Biotechnology, Inc. (Santa Cruz, CA, USA). Mouse anti-rat OX-42 (CD11b) antibody was obtained from AbD Serotec (Oxford, Canterbury, UK). Artemisinin was purchased from Guilin Pharmaceutical Factory (Guilin, Guangxi, P. R. China) and was dissolved in DMSO (final concentration <0.1% v/v).

### Primary microglial cell culture

Sprague-Dawley rats were obtained from the Experimental Animal Center of Sun Yat-sen University (Guangzhou, China). Experiments were carried out according to the National Institutes of Health Guide for Care and Use of Laboratory Animals and were approved by the Bioethics Committee of Sun Yat-sen University. Primary microglia were cultured from postnatal day 1 Sprague-Dawley rats as previously described [35]. Briefly, cerebral cortical fragments were dissociated by soft trituration in ice-cold DMEM containing 10% FBS, 2 mM glutamine, 100 IU/mL penicillin, and 100 µg/mL streptomycin. The cortical fragments were resuspended in culture medium and made into a single cell suspension by repeated pipetting. Single cells were plated on 100-mm culture dishes and incubated at 37°C in humidified 5% CO_2_/95% air for 2 weeks. The microglia were detached from the flasks by mild shaking and applied to a nylon mesh to remove astrocytes and cell clumps. Isolated microglia were plated on 24-well plates at a density of 2×10^5^ cells/well, and cultures with >90% purity of microglia (identified by an OX-42-specific antibody) were used. The cells were cultured for 2 days before drug treatment.

### BV-2 microglial cell culture

BV-2 cells were maintained in DMEM with 10% fetal bovine serum FBS in a 5% CO2 incubator. Plated cells were grown in DMEM with 10% FBS overnight. BV-2 cells were stimulated with 100 ng/ml LPS, 10 µM of artemisinin, or DMSO as control for 6 h. These stimulation conditions were adapted from previously published experiments [36].

### Cell viability assay

Cell viability was determined by the tetrazolium salt 3-[4,5-dimethylthiazol-2-yl] -2,5-diphenyltetrazolium bromide (MTT, Sigma-Aldrich) assay. Briefly, cells were seeded and treated with LPS (1 µg/mL) and various concentrations of artemisinin (ranging from 1 to 20 µM) for 1 h. The cells were also treated with 10 µM artemisinin and 1 µg/ml LPS for 6 to 48 h. After incubation for the indicated time, 0.5 mg/ml MTT was added to each well. The dark blue formazan crystals that were produced were dissolved in acidified isopropanol, and formazan quantification was performed at a test wavelength of 570 nm and a reference wavelength of 620 nm. Each experiment was performed in triplicate.

### siRNA transfection

The siRNA sequences that were used to silence IκB-α (GenBank NM_001105720) were designed and synthesized by Guangzhou Ribobio (Guangzhou, China): sense, 5′-CCAUGGAAGUGAUUGGUCAGGUGAA-3′; antisense, 5′-UUCACCUGACCAAUCACUUCCAUGG-3′. For nonsense control siRNA, we used irrelevant siRNA with random nucleotides that had no known specificity: sense, 5′-CCAAAGGUGUUAUGGGACUGUGGAA-3′; antisense, 5′-UUCCACAGUCCCAUAACACCUUUGG-3′. Microglia (1×10^6^) were plated in a 6-well plate, incubated for 48 h, and transfected with an IκB-α-specific siRNA duplex (50 nM final concentration) or the nonsense control siRNA (50 nM) using the Lipofectamine 2000 reagent (Invitrogen, Carlsbad, CA) according to the manufacturer's instructions. The transfected cells were stimulated with LPS (1 µg/ml) or artemisinin for 48 h.

### Nitrite assay

The production of NO was assayed by measuring the levels of nitrite, a stable NO metabolite, in the culture medium. Accumulation of nitrite in the medium was determined by a colorimetric assay with the Griess reagent. The supernatant was collected and mixed with an equal volume of Griess reagent (0.1% N-1-naphthylethylenediamine dihydrochloride and 1% sulphanilamide in 5% phosphoric acid) in a 96-well plate and incubated for 10 min at room temperature in the dark. Nitrite concentrations were determined using standard solutions of sodium nitrite prepared in cell culture medium. Absorbance was measured at 550 nm in a microplate reader.

### Transwell migration assay

Costar Transwell polycarbonate filters (8-µm pore size) were used in a migration assay to examine the ability of microglia migration. 1×10^6^ BV-2 cells in 1.5 ml serum-free medium were added to the upper well, and 2.6 ml serum-free medium was added to the lower chamber. 100 ng/ml LPS, 10 µm artimisinin, 100 ng/ml LPS + 10 µm artimisinin, or DMSO as solvent control were added to the lower chamber medium. At the end of a 24 h incubation period, cells that had migrated to the lower surface were quantified by counting the migrated cells on the lower surface of the membrane using microscopy.

### Western blot and quantitative real-time RT-PCR

Cells were pretreated with artemisinin prior to incubation with LPS. The cells were rinsed twice with ice-cold PBS and incubated in 0.5 ml of ice-cold lysis buffer for 20 min on ice. After the incubation, the cells were scraped and centrifuged. Cells were lysed in RIPA lysis buffer, and the protein concentrations were determined using the BCA protein assay (Pierce, Rockford, IL, USA). Equal amounts of protein were solubilized in Laemmli buffer, boiled for 5 min, separated by SDS-polyacrylamide gel electrophoresis and transferred to nitrocellulose membranes. Primary antibodies for IκB-α and phosphorylated-IκB-α (Ser32) were diluted 1∶500 in Tris-buffered saline with Tween (TBS-T) containing 5% nonfat milk, and the membranes were probed with the antibodies at 4°C overnight. After incubation with the primary antibody, the membranes were incubated with the appropriate secondary antibodies for 1 h at room temperature. Immunoreactive bands were visualized by an enhanced chemiluminescence (ECL, Amersham Pharmacia Biotech, Piscataway, NJ, USA) reaction.

For real-time reverse transcription PCR, total RNA was extracted from induced cell cultures using TRIzol (Invitrogen), and cDNA was synthesized using primers with the Advantage RT-for-PCR kit (BD Biosciences). We quantified the PCR amplifications using SYBR Green PCR Master Mix (Applied Biosystems), and the results were normalized to GAPDH gene expression. All of the experiments were performed in triplicate and repeated at least three times.

### Electrophoretic mobility shift assay

To examine the DNA binding activity of NF-κB, electrophoretic mobility shift assays (EMSAs) were performed according to the manufacturer's instructions (Gel Shift Assay System E3300, Promega, Madison, WI). Nuclear proteins were isolated as previously described [37], and protein concentrations were determined using the BCA protein assay with bovine serum albumin (BSA) as a standard.

### Statistical analysis

The results are presented as the means ± S.E.M, and one-way analysis of variance (ANOVA) was used to test the significance between groups. The Student's t test was used for the comparison of experimental groups in cell migration assays. Differences were considered significant at P<0.05.
